# Supplementary material for: Host-pathogen coevolution increases genetic variation in susceptibility to infection
Source: eLife. 2019 Apr 30;8:e46440. doi: 10.7554/eLife.46440 (PMC6491035; doi:10.7554/eLife.46440)
Supplement: Supplementary file 1. — The natural virus for each host is in red and bold. Genetic variances are estimated from the among-family variances in viral load. 95% CIs show differences in estimates of genetic variation for different host-virus combinations, intervals that do not cross zero represent statistically significant differences. [file elife-46440-supp1.docx]

| **Host** | **Virus comparison** | **95% CIs** |
| --- | --- | --- |
| *D. affinis* | **DAffSV**-DImmSV  **DAffSV**-DMelSV  DImmSV-DMelSV | 0.862 1.699  0.532 1.510  -0.606 0.004 |
| *D. immigrans* | **DImmSV**-DMelSV  **DImmSV**-DObsSV  DMelSV-DObsSV | 0.630 1.331  0.500 1.240  -0.365 0.167 |
| *D. melanogaster* | **DMelSV**-DAffSV  **DMelSV**-DObsSV  DAffSV-DObsSV | 0.080 0.485  0.012 0.486  -0.264 0.149 |
| *D. obscura* | **DObsSV**-DAffSV  **DObsSV**-DMelSV  DAffSV-DMelSV | 3.219 6.184  2.786 5.817  -0.996 0.102 |

**Table S1. Credible intervals of the differences between estimates of genetic variance in susceptibility across host species and viruses.** The natural virus for each host is in red and bold. Genetic variances are estimated from the among-family variances in viral load. 95% CIs show differences in estimates of genetic variation for different host-virus combinations, intervals that do not cross zero represent statistically significant differences.
