# Supplementary material for: Host-pathogen coevolution increases genetic variation in susceptibility to infection
Source: eLife. 2019 Apr 30;8:e46440. doi: 10.7554/eLife.46440 (PMC6491035; doi:10.7554/eLife.46440)
Supplement: Supplementary file 2. — The natural virus for each host is in red and bold. Genetic variances are estimated from the among-family variances in viral load. [file elife-46440-supp2.docx]

| **Host** | **Virus** | **Mean** | **95% CIs** | |
| --- | --- | --- | --- | --- |
| *D.affinis* | **DAffSV** | 1.47 | 1.12 | 1.92 |
| *D.affinis* | DImmSV | 0.18 | 0.05 | 0.32 |
| *D.affinis* | DMelSV | 0.48 | 0.21 | 0.77 |
| *D.immigrans* | **DImmSV** | 1.08 | 0.81 | 1.39 |
| *D.immigrans* | DMelSV | 0.11 | 0.00 | 0.27 |
| *D.immigrans* | DObsSV | 0.21 | 0.00 | 0.41 |
| *D.melanogaster* | **DMelSV** | 0.55 | 0.39 | 0.73 |
| *D.melanogaster* | DAffSV | 0.28 | 0.16 | 0.39 |
| *D.melanogaster* | DObsSV | 0.33 | 0.15 | 0.48 |
| *D.obscura* | **DObsSV** | 5.84 | 4.39 | 7.23 |
| *D.obscura* | DAffSV | 1.07 | 0.75 | 1.44 |
| *D.obscura* | DMelSV | 1.52 | 1.01 | 1.98 |

**Table S2. Estimates of genetic variation for each host virus combination.** The natural virus for each host is in red and bold. Genetic variances are estimated from the among-family variances in viral load.
