# Supplementary material for: Host-pathogen coevolution increases genetic variation in susceptibility to infection
Source: eLife. 2019 Apr 30;8:e46440. doi: 10.7554/eLife.46440 (PMC6491035; doi:10.7554/eLife.46440)
Supplement: Supplementary file 3. — The coevolved (natural) virus for each host is in red and bold. Models ran using REML gave similar estimates. [file elife-46440-supp3.docx]

| **Experiment** | **Host** | **Viruses** | ***r_g_*** | **95% CI** |
| --- | --- | --- | --- | --- |
| Full sib analysis | *D. affinis* | **DAffSV**-DImmSV  **DAffSV**-DMelSV  DMelSV-DImmSV | 0.50  0.05  0.02 | -0.01, 0.92  -0.54, 0.52  -0.62, 0.57 |
| Full sib analysis | *D. immigrans* | **DImmSV**-DMelSV  **DImmSV**-DObsSV  DMelSV-DObsSV | -0.32  -0.22  0.29 | -0.91, 0.33  -0.70, 0.26  -0.43, 0.94 |
| Full sib analysis | *D. melanogaster* | **DMelSV**-DAffSV  **DMelSV**-DObsSV  DAffSV-DObsSV | 0.40  0.25  0.36 | 0.20, 0.61  -0.02, 0.48  0.10, 0.62 |
| Full sib analysis | *D. obscura* | **DObsSV**-DAffSV  **DObsSV**-DMelSV  DAffSV-DMelSV | 0.21  0.40  0.68 | -0.03, 0.46  0.16, 0.63  0.49. 0.84 |
| DSPR QTL Mapping Population | *D. melanogaster* | **DMelSV**-DAffSV  **DMelSV**-DObsSV  DAffSV-DObsSV | 0.36  -0.07  0.10 | 0.21, 0.50  -0.21, 0.05  -0.06, 0.27 |

**Table S3. Genetic correlations (*r_g_*) in viral loads across host species after infection by coevolved and non-coevolved viruses** The coevolved (natural) virus for each host is in red and bold. Models ran using REML gave similar estimates.
