# Supplementary material for: Host-pathogen coevolution increases genetic variation in susceptibility to infection
Source: eLife. 2019 Apr 30;8:e46440. doi: 10.7554/eLife.46440 (PMC6491035; doi:10.7554/eLife.46440)
Supplement: Supplementary file 4. — PCRs were carried out using a touchdown PCR cycle (95°C 30sec, 62°C (-1°C per cycle) 30sec, 72°C 1min; for 10x cycles followed by; 95°C 30sec, 52°C 30sec, 72°C 1min; for a further 25x cycles). [file elife-46440-supp4.docx]

| **Primer** | **Sequence (5’-3’)** | **Primer information** |
| --- | --- | --- |
| Ge-1_Indel_1F  Ge-1_Indel_1R  Ref2p_P1_F  Ref2p_PS1_R  Ref_a1_F  Ref_a1_R  CHK_F  Doc1420_F  CHK_R | AGCGTCAAGCTTTTCCTTCA  CACCAGCGGTCAGGATAGAT  CTCACCCAGCTGCACTTGTA  TGTTGCAATCTTTGCGACTC  GGATGCCCTCCCAGAATTA  CGACGCAATRYGGTGTATCC  CTCTTGGCTCCAAACGTGAC  CTTGTTCACATTGTCGCTGAG  AAGGCAAACGACGCTCTT | *Ge-1* genotyping primers. Product with susceptible allele is 155bp and 77bp with resistance allele.  *p62 (Ref(2)P)* genotyping primers. Multiplex all four primers. Susceptible allele produces a 235bp product, resistant allele a 289bp product.  *CHKov-1* genotyping primers. Use reverse primer (R) in singleplex with each of the forward (F) primers, if resistant Doc1420_F + CHK_R primers produce 560bp product, if susceptible CHK_F + CHK_R produce 634bp product |

**Table S4. Primers for genotyping *D. melanogaster* resistance genes *Ge-1, p62 (Ref(2)P) and CHKov-1.*** PCRs were carried out using a touchdown PCR cycle (95°C 30sec, 62°C (-1°C per cycle) 30sec, 72°C 1min; for 10x cycles followed by; 95°C 30sec, 52°C 30sec, 72°C 1min; for a further 25x cycles).
