# Supplementary material for: Host-pathogen coevolution increases genetic variation in susceptibility to infection
Source: eLife. 2019 Apr 30;8:e46440. doi: 10.7554/eLife.46440 (PMC6491035; doi:10.7554/eLife.46440)
Supplement: Supplementary file 5. [file elife-46440-supp5.docx]

**Table S5 QTL and their locations**

| QTL | Virus^1^ | Chromosome | Position^2^ | Map  Position | LOD | Confidence Interval^2,3^ |
| --- | --- | --- | --- | --- | --- | --- |
| X_13 | DMelSV | X | 5670000 | 13.3 | 14.2 | X:5530000..5740000 |
| 2L_53 | DMelSV | 2L | 19720000 | 53.7 | 23.9 | 2L:19540000..19870000 |
| 2R_100 | DMelSV | 2R | 18710000 | 100.9 | 11.5 | 2R:18650000..18770000 |
| 3L_46 | DMelSV & DObsSV^4^ | 3L | 20080000 | 46.1 | 33.5 | 3L:19990000..20140000 |
| 3R_49 | DMelSV & DObsSV^5^ | 3R | 5550000 | 49 | 21.3 | 3R:5420000..5930000 |
| 3R_64 | DMelSV | 3R | 14960000 | 64.9 | 13.4 | 3R:14900000..15060000 |
| 3R_69 | DMelSV | 3R | 16640000 | 69.6 | 10.8 | 3R:16570000..16840000 |

^1^ The virus used to identify the QTL

^2^ Release 5 of the *D. melanogaster* genome

^3^ Confidence interval on location based on a LOD drop of 2

^4^ QTL significant for both DMelSV and DObsSV, but showing location from DObsSV

^4^ QTL significant for both DMelSV and DObsSV, but showing location from DMelSV
