# Supplementary material for: Host-pathogen coevolution increases genetic variation in susceptibility to infection
Source: eLife. 2019 Apr 30;8:e46440. doi: 10.7554/eLife.46440 (PMC6491035; doi:10.7554/eLife.46440)
Supplement: Supplementary file 6. — RpL32 primers overlap an intron-exon boundary. Sigma virus primers cross gene boundaries except for DImmSV that amplifies the L gene [file elife-46440-supp6.docx]

| **Primers** | **Forward** | **Reverse** |
| --- | --- | --- |
| D.mel_*RpL32* | TGCTAAGCTGTCGCACAAATGG | TGCGCTTGTTCGATCCGTAAC |
| D.imm_*RpL32* | TGCTAAGTTGTCGCACAAATGG | TACGCTTGTTGGAGCCATAAC |
| D.aff_*RpL32* | TGCCAAGTTGTCGCACAAATGG | TGCGCTTGTTGGAGCCATAAC |
| D.obs_*RpL32* | TGCTAAGTTGTCGCACAAATGG | TGCGCTTGTTGGAACCGTAAC |
| DMelSV | CCGACTACAAATGCTATATG | CAGGTATTAGAGGCTTCTTA |
| DImmSV | CAGAGTATTTGATCAAGGAG | TGGCAATACAGATGTTTTG |
| DAffSV | CCAGGTGACTAACGATTC | GGAAGAGGAGATATTCAAG |
| DObsSV | GGCATCTGTTACCGTTAA | ACTCGGAATTTTCTGACC |

**Table S6. Primers for qRT-PCR (5’-3’).** RpL32 primers overlap an intron-exon boundary. Sigma virus primers cross gene boundaries except for DImmSV that amplifies the L gene
